# Itraconazole Nanosuspensions via Dual Centrifugation Media Milling: Impact of Formulation and Process Parameters on Particle Size and Solid-State Conversion as Well as Storage Stability

**DOI:** 10.3390/pharmaceutics14081528

**Published:** 2022-07-22

**Authors:** Ann-Cathrin Willmann, Kai Berkenfeld, Thilo Faber, Herbert Wachtel, Georg Boeck, Karl G. Wagner

**Affiliations:** 1 Pharmaceutical Development, Boehringer Ingelheim Pharma GmbH & Co. KG, Birkendorfer Straße 65, 88397 Biberach, Germany; ann-cathrin.willmann@boehringer-ingelheim.com; 2 Department of Pharmaceutical Technology, University of Bonn, Gerhard-Domagk-Straße 3, 53121 Bonn, Germany; kaib@uni-bonn.de (K.B.); thilo.faber@uni-bonn.de (T.F.); 3 Device Development, Boehringer Ingelheim GmbH & Co. KG, Binger Straße 173, 55216 Ingelheim am Rhein, Germany; herbert.wachtel@boehringer-ingelheim.com; 4 Department Discovery Research, Boehringer Ingelheim RCV GmbH & Co. KG, Dr.-Boehringer-Gasse 5-11, 1121 Vienna, Austria; georg.boeck@boehringer-ingelheim.com

**Keywords:** dual centrifugation, itraconazole, media milling, nanocrystals, nanosuspension, agglomeration, amorphization

## Abstract

Nanocrystal suspensions proved to be a potent enabling principle for biopharmaceutics classification system class II drugs with dissolution limited bioavailability. In the example of itraconazole (ITZ) as a model drug combined with electrosteric stabilization using hydroxypropyl cellulose (HPC-SL), sodium dodecyl sulfate (SDS) and polysorbate 80 (PS80), the impacts of formulation and process parameters of a dual centrifugal mill on material attributes such as particle size, zeta potential, particle morphology, storage stability and especially solid-state characteristics were evaluated. A minimal concentration of 0.9% (*w*/*w*) HPC-SL, 0.14% (*w*/*w*) SDS and 0.07% (*w*/*w*) PS80 was necessary for sufficient nanoparticle stabilization. Despite the minor effect of PS80, its presence was beneficial for electrosteric stabilization. Choosing lower stabilizer concentrations resulted in a pronounced increase in particle size due to agglomeration, which was confirmed by SEM imaging and a decrease in zeta potential in combination with an amorphization of the particles. Milling temperature had no significant impact on the particle size, whereas milling speed and the size of the milling beads used were found to have a strong impact on the critical material attributes such as particle size and polydispersity index. The smallest particle sizes could be obtained by using the smallest milling bead size. However, the smallest obtainable particle size could only be achieved by using two-fold stabilizer concentrations, as smaller particles exhibit a larger specific surface area.

## 1. Introduction

The trend of an increasing number of poorly soluble compounds entering pharmaceutical development is still ongoing. It is reported that 40% of the drugs currently marketed and up to 75% of compounds under development are poorly water-soluble [1]. As an increase in surface area leads to a faster dissolution rate and a potentially higher kinetic solubility [2], one approach to enhance bioavailability is the reduction of particle size to generate nanosuspensions.

In the last few years, over 50 products that comprise nanoparticles have been approved by the FDA (U.S. Food and Drug Administration) [3]. In general, two different approaches, known as bottom-up (e.g., precipitation) or top-down (e.g., milling) processes, can be used to prepare nanosuspensions. Common top-down approaches are high-pressure homogenization and media milling. Media milling, which is also known as ‘wet stirred media milling’ or ‘bead milling’, decreases the size of particles suspended in a liquid by means of milling beads. Particle comminution is achieved by shear forces generated by movement of the stabilizer medium and beads, as well as collisions of API (active pharmaceutical ingredient)–API particles, API–wall or API–beads.

Upon milling, two different processes occur concurrently: the suspended particles break down due to mechanical stress, and, in parallel, might also agglomerate due to hydrophobic interparticulate forces (i.e., van der Waals forces) [4]. A decrease in particle size results in an increased surface area. As a consequence, the Gibbs free energy is increased, which leads to a thermodynamically unstable system that tends to reduce the free energy by particle agglomeration, flocculation or crystal growth [5], also known as Ostwald ripening [6,7]. To overcome these issues, a suitable stabilizer system is needed [8]. There are two main approaches to stabilize nanosuspensions, the steric stabilization by forming a physical barrier, e.g., by polymers, and/or the electrostatic stabilization by electrostatic repulsion. The latter mechanism is applicable for ionic stabilizers, e.g., sodium dodecyl sulfate (SDS). According to the DLVO (Deryaguin–Landau–Verwey–Overbeek) theory [9,10], the ionic surfactant enables the formation of an electric double layer on the surface of the nanoparticles, resulting in repulsive forces if particles approach each other [5]. The group of non-ionic stabilizers comprises polymers such as cellulose derivates (e.g., hydroxy propyl cellulose, HPC) and non-ionic surfactants, for example, polysorbates (PS). These stabilizers adsorb onto the surface of the particles and form a physical barrier towards the other particles in the suspension. The combined stabilization approach of both principles is known as electrosteric stabilization [4]. The composition and concentration of the stabilizer system is of utmost importance during the development of a nanosuspension formulation. In addition to the particle size, there are several other attributes that should be considered critical for the development of nanosuspensions, such as particle shape and morphology, solid-state properties, and chemical and physical stability. For investigating these parameters at material limited conditions during early formulation development, small scale milling approaches are required.

In this study, the preparation of nanosuspensions was carried out using a novel centrifugal mill that consists of a rotor with two sample adapters (ZentriMix; Andreas Hettich GmbH & Co. KG, Tuttlingen, Germany). Each sample adapter facilitates a secondary rotation (in addition to the rotor’s rotation), which is called dual centrifugation. This leads to a very high grinding energy. Further details concerning the ZentriMix can be found in an article by Hagedorn et al. [11]. Moreover, the rotor holds four samples to be processed simultaneously at a very small batch size of 5.5 g. As a biopharmaceutics classification system class II model compound, itraconazole was used because of its very low solubility of ~1 ng/mL in water and its high lipophilicity (logP~6) [12]. Several research groups have already investigated the application of media milling techniques to prepare ITZ nanosuspensions [13,14,15,16,17,18] utilizing agitator bead mills. This research was typically conducted with the aim of manufacturing intermediate scale batch sizes of 150–300 mL within a relatively short time frame (<2 h). On the contrary, the aim of the current study is to demonstrate the benefit of using a novel type of mill to accelerate early formulation development for nanosuspensions. We would like to highlight that, in early-stage formulation development, the ability to concurrently process multiple samples is a key factor to time- and cost-efficient formulation screening, so this type of mill, to our understanding, is a valuable addition to the techniques already established. The influence of process parameters such as processing time, milling speed, milling temperature and milling bead size was evaluated regarding particle size, polydispersity index (PDI) and the respective storage stability. Furthermore, the effect of the stabilizer concentration on critical material attributes of the ITZ nanosuspensions was examined. The grinding limit, which is the minimal achievable (i.e., constant) particle size during prolonged milling, was chosen as the primary endpoint in terms of particle size [19].

## 2. Materials and Methods

### 2.1. Materials

Itraconazole (ITZ) was purchased from Sigma-Aldrich (Supplier: Wuhan Atomole Chemicals Co., Ltd., Wuhan, China) and jet-milled prior to use. Hydroxypropyl cellulose (HPC-SL; Nippon Soda Co., Ltd., Tokyo, Japan), polysorbate 80 (PS80; Kolb Distribution AG, Hedingen, Switzerland) and sodium dodecyl sulfate (SDS; BTC Europe GmbH, Monheim am Rhein, Germany) were used as stabilizers for the nanosuspensions. Milling beads (zirconium oxide, yttrium stabilized, density 6 g/mL, diameter 0.3–0.4 mm (except for investigation of the effect of bead size, see Section 3.2.3)) were purchased from VMA Getzmann GmbH (Reichshof, Germany). For milling trials, 10 mL polypropylene (PP) vials from iphas (iphas Pharma-Verpackung GmbH, Würselen, Germany) were used.

### 2.2. Manufacturing Methods

#### 2.2.1. Preparation of ITZ Suspensions

For the stabilizer solution, surfactants (SDS and PS80) were dissolved in demineralized water, and HPC-SL was added and stirred overnight at 200 rpm with a magnetic stirrer to enable complete dissolution. To ensure a homogeneous suspension, 4.2 g of jet milled ITZ (d_10_ = 0.70 µm, d_50_ = 1.55 µm, d_90_ = 3.47 µm) and 42 g of the filtered (0.2 µm, cellulose acetate membrane) stabilizer solution were pre-suspended and mixed in a 100 mL duran glass bottle (filling level approximately 40% (*v*/*v*)) with the acoustic mixer LabRAM™ II (Resodyn Acoustic Mixers, Butte, MO, USA) at 50 times acceleration of gravity for two cycles of 45 s each. The mixer combined low oscillation frequencies at a resonant condition (approximately 60 Hz) with a relatively large displacement amplitude (up to 14 mm) to a high mixing energy, which was measured as the acceleration of gravity [20,21].

#### 2.2.2. Nanomilling by Dual Centrifugation

Nanomilling was performed using a ZentriMix 380R (Andreas Hettich GmbH & Co. KG, Tuttlingen, Germany) dual centrifuge. The rotor held four 10 mL PP processing vials, filled with 5.5 g of the homogenized suspension and 5 g of milling beads of various sizes (Table 1).

In order to determine the grinding limit, samples were taken in each run from the same vial every 30 min during the first 4 h, and afterwards every hour over a period of 8 h. The volume removed was replaced by fresh stabilizer solution.

For process evaluation, −10 °C and +4 °C were set as ambient temperature in the milling chamber. The temperature inside the vials was determined using a Testo 925 thermometer (Testo SE & Co. KGaA, Titisee-Neustadt, Germany). An overview of the parameters for the process evaluation can be found in Table 1. Assessing the smallest milling beads (0.1–0.2 mm), the minimum concentration of both stabilizers (SDS and PS80) had to be increased for this milling bead size only from 0.14% (*w*/*w*) to 0.28% (*w*/*w*) in order to get a stable nanosuspension (see Section 4).

#### 2.2.3. Storage Stability

The storage stability of the nanosuspensions was investigated for two out of six formulations (F2 and F6, see Table 2) at room temperature (22 °C ± 1 °C) and at refrigerated conditions for 7 and 14 days, respectively. Both formulations, milled for 3 h, were additionally put on long-term stability for 70 weeks at refrigerated conditions. The formulations were selected based on the results of the stabilizer assessment described in Section 3.1. F6 was selected as it yielded a constant particle size during milling, while minimizing the concentration of stabilizer needed. F2 was selected because it was the formulation with the lowest stabilizer concentration that resulted in a homogeneous suspension with the largest particle size.

### 2.3. Physicochemical Characterization Methods

#### 2.3.1. Particle Size Determination

A Zetasizer^®^ Nano S (Malvern Panalytical, Herrenberg, Germany) was used for the determination of the particle size distribution of the nanoparticles by dynamic light scattering. The nanosuspensions were diluted with filtered (0.2 µm, cellulose acetate membrane), saturated ITZ solution in demineralized water at a ratio of 1:500 [22]. The measurement was carried out with disposable semi-micro polymethyl methacrylate cuvettes at 25 °C. Detection angle was fixed at 173°. Ten measurements were performed per experiment and each sample was measured in triplicate. Particle size and size distribution were displayed as Z-average and PDI. Solution viscosity and refractive index were set to 0.8872 cP and 1.33, respectively. Particle size data of the nanoparticles were processed with Zetasizer^®^ Software Version 7.12 (Malvern Panalytical, Herrenberg, Germany).

#### 2.3.2. Zeta Potential

Zeta potential was determined with a SZ-100 nanoPartica analyzer (Horiba Europe GmbH, Oberursel, Germany). A 6 mm cuvette with a carbon electrode was used. The conductivity of the samples was adjusted as described in [23], and the temperature was kept at 25 °C. The sample was diluted 1:100 with filtered (0.2 µm, cellulose acetate membrane) demineralized water. The zeta potential was calculated (based on the electrophoretic mobility) using the Helmholtz–Smoluchowski equation with Horiba NextGen Project 2.4 software.

#### 2.3.3. X-ray Powder Diffractometry (XRPD)

After milling, the samples were centrifuged at 20,817 rcf (relative centrifugation force) for 15 min and the residue was used to investigate its solid-state properties. The diffraction patterns were obtained using a Bruker AXS D8 Discover DAVINCI diffractometer (Bruker Corporation, Billerica, MA, USA) using Cu-Kα radiation (40 kV voltage and 40 mA current). All scans were performed at 0.5 s/step with step size of 0.02° from 5° to 40° 2 Theta angles. Measurements were carried out in reflection mode (coupled two Theta/Theta). Raw XRPD data were converted to American Standard Code for Information Interchange (ASCII) format with Bruker DIFFRAC 7.5 and processed using Origin Pro^®^ 2017 (Origin Lab^®^ Corporation, Northampton, MA, USA).

#### 2.3.4. Differential Scanning Calorimetry (DSC)

The thermal behavior of the nanosuspensions was investigated by differential scanning calorimetry with a DSC2500 Discovery (TA Instruments^®^, New Castle, DE, USA). The samples used for X-ray diffractometry were reused in order to save material and dried at 40 °C under vacuum for 1 h to remove any residual water. Then, 3–5 mg were weighed out into aluminum pans with a perforated lid and compacted gently with a punch in order to ensure a homogeneous heat transfer. Nitrogen was used as a purging gas at a flow rate of 50 mL/min. Samples were heated at 10 °C/min from 25 °C to 200 °C. DSC data were analyzed using TA Instruments TRIOS software (TA Instruments, New Castle, DE, USA).

#### 2.3.5. Scanning Electron Microscopy (SEM)

The nanosuspensions were diluted 1:500 with demineralized water and dried over night at room temperature on a coverslip. A Helios™ G4 CX Dualbeam™ (Thermo Scientific™ Inc, Waltham, MA, USA) microscope was used in secondary electron mode at 2 kV and 3 mm working distance. Samples were sputtered with platinum.

### 2.4. Statistical Evaluation

Results were expressed as mean ± standard deviation (SD). The effect of process parameters (bead size, temperature, speed) on the particle size was investigated by a one-way ANOVA with post hoc Tukey test. A paired-sample *t*-test was applied for the investigation of storage stability. Comparison of the two selected formulations in terms of particle size, PDI and zeta potential at specific time points was done by a two-sample *t*-test. Statistical evaluation of the stabilizer concentration was performed using MODDE^®^ 12.1 software (Sartorius Stedim Biotech GmbH, Göttingen, Germany). All other statistical investigations and data processing were carried out with Origin Pro 2017 (Origin Lab^®^ Corporation, Northampton, MA, USA). *p* < 0.05 was considered to represent a significant difference.

## 3. Results

The evaluation of process parameters was performed at fixed formulation conditions; vice versa, experiments regarding the formulation impact on critical material attributes of the nanosuspension were carried out at fixed process parameters. An overview of the respective settings is given in Table 1.

### 3.1. Stabilizer Assessment

To optimize the process of nanomilling, binary and ternary stabilizer mixtures of HPC-SL, SDS and PS80 were evaluated by means of a design of experiments approach. A response surface model (RSM) was applied and planned as a full factorial design with two factors (k: SDS and PS80) at three concentration levels. The resulting number of experiments is 3^k^ = 9. Three repetitions per experiment resulted in a total number of 27 experiments. As three combinations did not yield a stable suspension, only 27 − 3 × 3 = 18 trials were evaluated. The trials were conducted with the following parameters: bead size 0.3–0.4 mm, milling temperature 4 °C and centrifugation speed of 1500 rpm.

**Figure 1:**
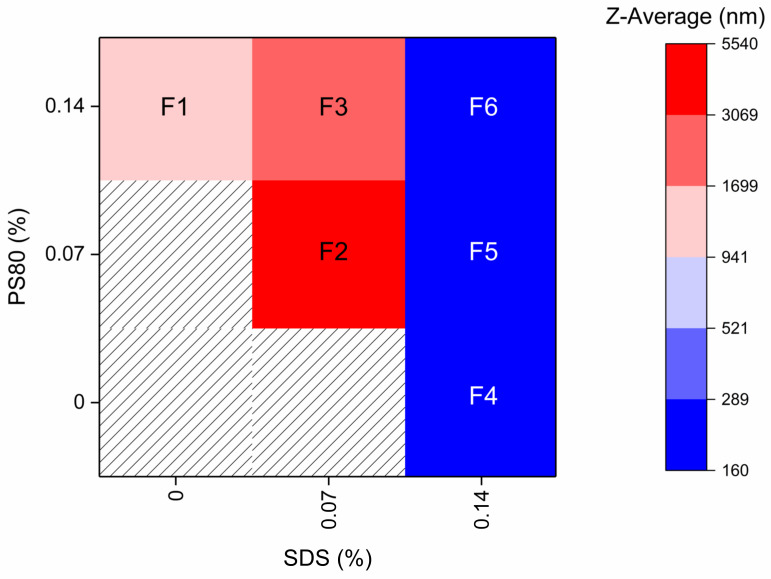
Z-average after 8 h of milling with different stabilizer solutions. Composition is given in % (*w*/*w*) and HPC-SL concentration was kept constant at 0.9%. Milling was carried out at 4 °C with 1500 rpm and milling beads sized 0.3–0.4 mm. Hatched area: No stable nanosuspension was obtained. Values are given as mean of three measurements.

The concentration of HPC-SL was kept constant at 0.9% (*w*/*w*) for all formulations (F1–F6), as previous tests showed no improvement when using higher concentrations (data not shown). ITZ concentration was also kept constant at 7.4% (*w*/*w*) for all formulations after 8 h of milling. The input variables and the response parameters are reported in Table 2. The primary response parameter, particle size, reported in Figure 1, was obtained after 8 h of milling. The hatched areas represent compositions resulting in inhomogeneous nanosuspensions, which were neglected for statistical evaluations. All suspensions with a SDS concentration of 0.07% (*w*/*w*) or less (F1–F3) were neither homogeneous nor of a particle size below 235 nm.

The smallest Z-average in the steady range of 160–170 nm was found with 0.14% (*w*/*w*) SDS and 0.07 to 0.14% (*w*/*w*) PS80 (F4–F6).

The summary of fit for the model is presented in Table 3. Analysis of the coefficients showed that the concentration of SDS had the highest impact on particle size, i.e., led to a smaller particle size (*p* < 0.001). The PS80 concentration also had a significant effect on the particle size (*p* < 0.001), but to a lower extent when compared to the concentration of SDS.

Furthermore, a weak interaction between the concentration of SDS and PS80 was observed. With the unscaled coefficients, the resulting particle size could be calculated depending on the SDS and PS80 concentration, as shown in Equation (1) (applicable in a concentration range of 0% (*w*/*w*)–0.14% (*w*/*w*) each):

(1) $$\begin{array}{cc} Z-\mathrm{Average} & =K+c_{\mathrm{SDS}}\;\times \;k_{\mathrm{SDS}}+c_{\mathrm{PS}80}\;\times \;k_{\mathrm{PS}80}+(c_{\mathrm{SDS}}{)}^{2}\;\times \;k_{\mathrm{SDS}\times \mathrm{SDS}\;}+k_{\mathrm{SDS}}\;\times \;c_{\mathrm{PS}80}\;\times \;k_{\mathrm{SDS}\times \mathrm{PS}80}\\ & =13463.2+c_{\mathrm{SDS}}\;\times \;\left(-51352.6\right)+c_{\mathrm{PS}80}\;\times \;\left(-85962.2\right)+(c_{\mathrm{SDS}}{)}^{2}\;\times \;(-309054)+c_{\mathrm{SDS}}\;\times \;c_{\mathrm{PS}80}\;\times \;610912 \end{array}$$

where K is a constant, $c_{\mathrm{SDS}}$ is the concentration of SDS [% *w*/*w*], $k_{\mathrm{SDS}}$ is the coefficient for concentration of SDS, $c_{\mathrm{PS}80}$ is the concentration of PS80 [% *w*/*w*], $k_{\mathrm{PS}80}$ is the coefficient for concentration of PS80, $k_{\mathrm{SDS}\times \mathrm{SDS}}$ is the coefficient for concentration of SDS in a quadratic term and $k_{\mathrm{SDS}\times \mathrm{PS}80}$ is the coefficient for the interaction between the concentration of SDS and the concentration of PS80.

Subsequent sampling during the analysis led to a decrease in the concentration of ITZ from 9.1% (*w*/*w*) to 7.4% (*w*/*w*) after 8 h of milling as suspension samples withdrawn were replaced with fresh stabilizer medium. F5 und F6 were tested in a follow-up study without sampling (9.1% (*w*/*w*) concentration of ITZ): The combination of 0.14% (*w*/*w*) SDS and 0.07% (*w*/*w*) PS80 (F5) was suitable for stabilizing a nanosuspension comprising 7.4% (*w*/*w*) ITZ, but not the initial concentration of 9.1% (*w*/*w*) (data not shown). Therefore, F6 with 0.9% (*w*/*w*) HPC-SL, 0.14% (*w*/*w*) SDS and 0.14% (*w*/*w*) PS80 was chosen as the optimum composition to stabilize the ITZ nanosuspension.

### 3.2. Evaluation of Milling Process Parameters

For the evaluation of the milling process parameters, formulation F6 was used.

#### 3.2.1. Effect of Milling Intensity on the Particle Size

In order to evaluate the impact of the milling intensity, the maximum reasonable settings for this rotor (500 rpm and 1500 rpm) were tested. Using various milling speeds led to significantly different particle sizes: after 3 h of milling at 500 rpm, the Z-average of the ITZ particles was reduced to 275 nm ± 1 nm, whereas milling at 1500 rpm resulted in a Z-average of 166 nm ± 1 nm (*p* < 0.001). This effect was even more pronounced after 8 h of milling, resulting in 201 nm ± 1 nm at 500 rpm and 164 nm ± 2 nm at 1500 rpm (*p* < 0.001), respectively. Milling at 1500 rpm was preferred because of the reduced process time and the smaller Z-average obtained.

#### 3.2.2. Effect of Milling Temperature on the Particle Size

Two milling temperatures were tested: 4 °C (which was the manufacturer’s recommendation) and −10 °C, which was the lowest temperature supported by the centrifuge. The above-mentioned temperatures were set for the milling chamber, whereas the temperatures inside the vials were higher because of the process heat, yet still constant during the milling process at 25.6 °C ± 0.2 °C for milling at 4 °C and 16.3 °C ± 0.4 °C for milling at the set temperature of −10 °C, respectively. No degradation occurred during milling at 4 °C (determined via HPLC, data not shown). The particle sizes obtained at different vial temperatures were similar with 166 nm ± 1 nm after 3 h at a vial temperature of approximately 26 °C and 169 nm ± 2 nm for a vial temperature of approximately 16 °C, respectively.

#### 3.2.3. Effect of Bead Diameter on the Particle Size

**Figure 2:**
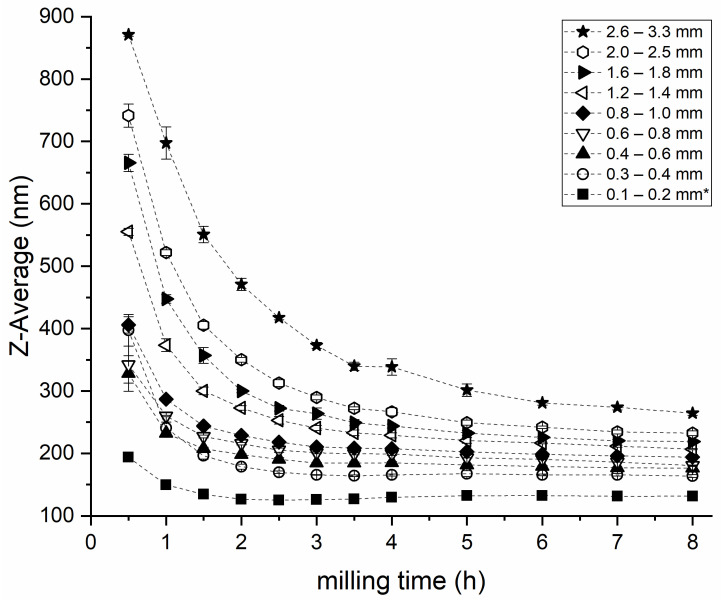
Effect of milling bead size on the ITZ particle size. Values are expressed as mean ± SD, *n* = 3. Milling was carried out at 4 °C with 1500 rpm. * Bead size was 0.1–0.2 mm; stabilizer concentration had to be changed to 0.9% (*w*/*w*) HPC-SL, 0.28% (*w*/*w*) PS80 and 0.28% (*w*/*w*) SDS (see Section 4).

Figure 2 depicts the Z-average of the ITZ nanoparticles generated with different bead diameters as a function of the milling time, using 0.14% (*w*/*w*) SDS and 0.14% (*w*/*w*) PS80, except for the lowest bead size. The impact of different sized beads was evaluated for nine different bead diameters, as shown in Table 4.

The theoretical number n of 5 g beads (e.g., diameter 0.3–0.4 mm) can be calculated according to Equation (2):

(2) $$n\;=\;\frac{V_{5g}}{V_{0.35}}\;=\;\frac{833.33\;{\mathrm{mm}}^{3}}{0.022449\;{\mathrm{mm}}^{3}}\;=\;37121$$

where $V_{5g}$ is the volume of 5 g beads calculated with the bead density of 6 g/cm^3^ and $V_{0.35}$ is the volume of one bead, assuming a mean diameter of 0.35 mm.

Milling with all nine bead sizes led to stable and homogeneous nanosuspensions. The lowest achievable particle size after 8 h of milling was directly correlated to the grinding bead diameter. The smallest particle size was achieved with the smallest milling beads (diameter 0.1–0.2 mm, Figure 2) However, the smaller beads demanded twice the stabilizer concentration used with larger milling beads to achieve constant particle sizes (see Section 4).

Using twice the concentration of SDS (0.28% *w*/*w*) and PS80 (0.28% *w*/*w*) in combination with the smallest milling beads (0.1–0.2 mm), a stable nanosuspension with a particle size of 127 nm ± 1 nm was generated with 0.1–0.2 mm beads after 3 h of milling with a corresponding PDI of 0.18 ± 0.01. The largest beads (2.6–3.3 mm) led to the nanosuspension with the largest particle size and the greatest PDI after 8 h of milling: 264 nm ± 4 nm and 0.26 ± 0.00, respectively; however, at a stabilizer concentration of 0.14% each. All samples showed significantly different particle sizes after the end of the milling process (*p* < 0.001), except for the samples milled with 0.4–0.6 mm and 0.6–0.8 mm, which resulted in a particle size of 176 nm ± 3 nm and 181 nm ± 1 nm (*p* = 0.64), respectively. The PDI of the samples milled with beads of 1.2–1.4 mm and smaller decreased at the beginning of the milling process (as shown in Figure S1) and rose only slightly during prolonged milling. On the contrary, milling beads of 1.6–1.8 mm and larger resulted in an initial increase of the PDI and a subsequent drop at about the same time the minimum particle size/grinding limit was reached. More details can be found in Figure S1 in the Supplementary Materials.

For the determination of the grinding limit, samples were compared after a milling time of 6 h and 8 h (see Figure 2). The particle sizes of the suspensions generated with 0.1–0.2 mm to 0.4–0.6 mm beads were not significantly different at these time points (0.1–0.2 mm: *p* = 0.75, 0.3–0.4 mm: *p* = 0.29, 0.4–0.6 mm: *p* = 0.19), and thus the grinding limit was reached. Regarding the suspensions milled with milling bead diameters of 0.6–0.8 mm and greater, the grinding limit was not reached within the investigated timeframe (*p* < 0.001, except for 0.8 mm–1.0 mm: *p* = 0.03 and 2.0–2.5 mm: *p* = 0.03). The smallest particle size with the least stabilizer concentration was achieved with the bead size 0.3–0.4 mm.

### 3.3. Impact of Concentration of Stabilizer on CMA

A comparison of F6 vs. F2 was performed to investigate the impact of the stabilizer concentration on the critical material attributes. F2 was chosen as this was the formulation with the lowest stabilizer concentration that resulted in a homogeneous suspension with the greatest particle size, and F6 as the formulation with a constant particle size during milling with a stabilizer concentration as low as possible.

#### 3.3.1. Impact of Concentration of Stabilizer on Z-Average, PDI and Zeta Potential

**Figure 3:**
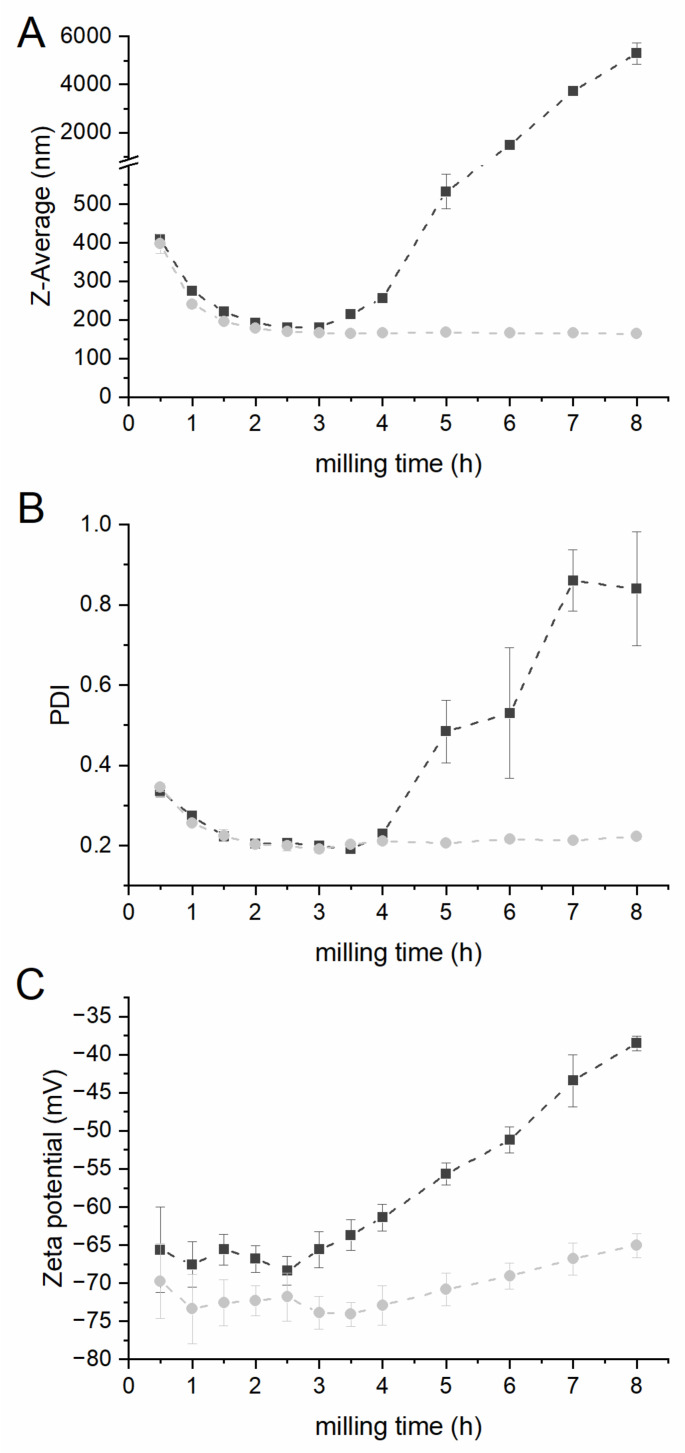
Z-Average (**A**), PDI (**B**) and zeta potential (**C**) of F2 (black squares) and F6 (grey dots) in relation to the milling time. Milling was carried out at 4 °C with 1500 rpm and milling beads sized 0.3–0.4 mm. Values expressed as mean ± SD, *n* = 3.

Figure 3 displays the Z-average (A), the PDI (B) and the zeta potential (C) for both formulations, F2 and F6, in relation to the milling time. Significant changes in particle size, PDI and zeta potential were observed after 2.5 h of milling for formulation F2. After the first 2.5 h of milling, the particle size decreased to 181 nm ± 1 nm, and afterwards increased to 5297 nm ± 441 nm with no final grinding limit (*p* < 0.001). The zeta potential values increased after milling for 2.5 h of milling, and afterwards decreased significantly from |−68| mV ± 2 mV to |−39| mV ± 1 mV after 8 h of milling (*p* < 0.001). After a process time of 2.5 h, the PDI was found to be 0.20 ± 0.01, but increased to 0.84 ± 0.14 after milling for 8 h (*p* < 0.001). On the contrary, the particle size of formulation F6 remained constant after 3 h of milling, thus the grinding limit of this formulation (166 nm ± 1 nm) was reached. The PDI exhibited a statistically significant increase from 0.19 ± 0.01 to 0.22 ± 0.01 after 3 h and 8 h of milling (*p* = 0.01), respectively. This was in correspondence with the decrease in zeta potential value from |−74.1| mV ± 1.6 mV to |−65| mV ± 1.6 mV after 8 h (*p* = 0.01).

#### 3.3.2. Impact of Concentration of Stabilizer on the Solid-State Properties

**Figure 4:**
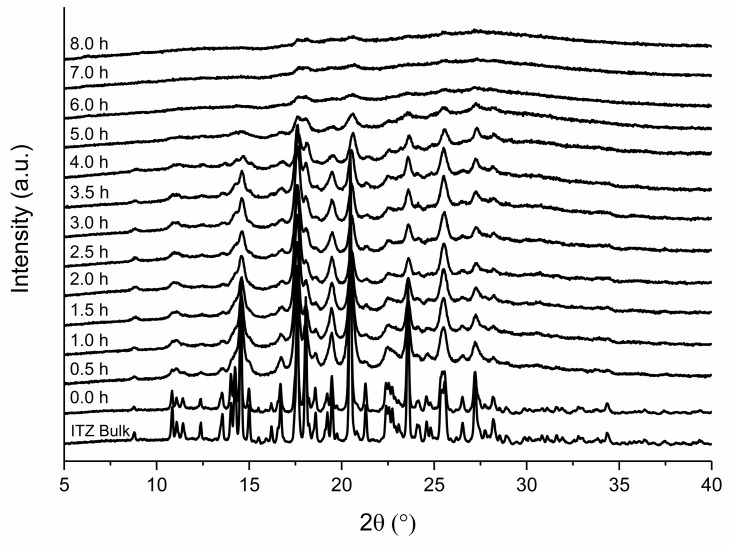
XRPD pattern of F2 in relation to grinding time. Milling was carried out at 4 °C with 1500 rpm and milling beads sized 0.3–0.4 mm. Plots are shifted for a better illustration.

Figure 4 illustrates the XRPD patterns of formulation F2 after different milling times. The unprocessed ITZ suspension showed distinct peaks of ITZ, which correspond to the ITZ bulk material. A reduction in peak intensity, as well as peak broadening, occurred during the milling process and was observed already at the first time point after 0.5 h of milling. After 8 h of milling, the peaks indicating crystalline structures were no longer present. As an example, the signal-to noise ratio of the peak at 23.56° decreased from 2 (after 0.5 h) to 1 (after 8 h of milling).

**Figure 5:**
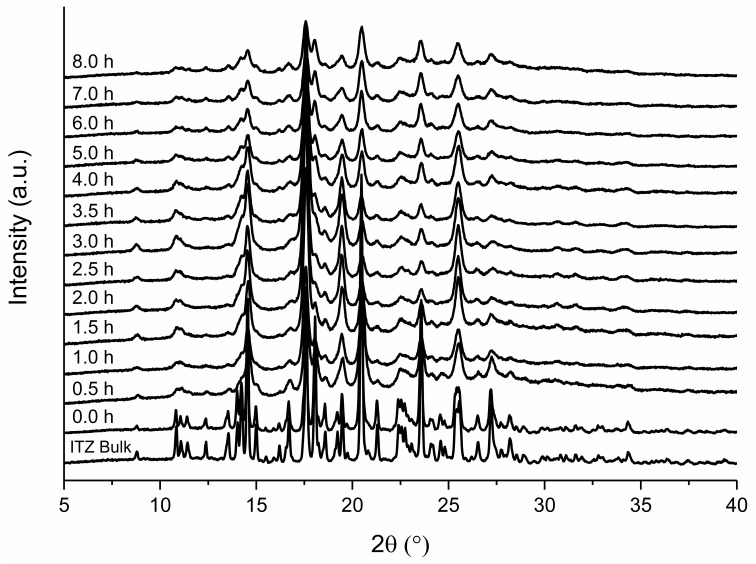
XRPD pattern of F6 in relation to grinding time. Milling was carried out at 4 °C with 1500 rpm and milling beads sized 0.3–0.4 mm. Plots are shifted for a better illustration.

A slightly different result was found for F6 (Figure 5). In addition, formulation F6 exhibited a reduction in peak intensity as well as peak broadening after 0.5 h of milling. However, in contrast to formulation F2, the peak intensity did not decrease to the same extent and was still indicating crystalline material after 8 h of milling. The signal noise ratio of the peak at 23.56° stayed constant at 2 and the peaks remained clearly present.

#### 3.3.3. Impact of Concentration of Stabilizer on the Thermal Behavior

**Figure 6:**
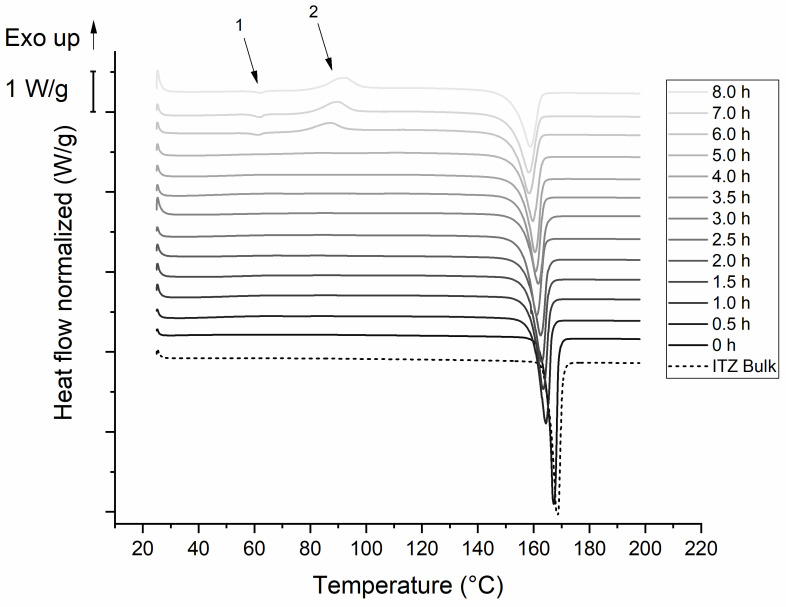
DSC-thermograms of F2 ITZ nanosuspension. Milling was carried out at 4 °C with 1500 rpm and milling beads sized 0.3–0.4 mm. Plots are shifted for a better illustration. Arrow 1 marks an endothermic event, arrow 2 marks an exothermic event.

Figure 6 shows DSC-thermograms of formulation F2 obtained after the indicated time points during the milling process. An endothermic event at approximately 62 °C occurred after 6 h of milling (marked with arrow 1). It was followed by an exothermic event with an onset temperature of 87–90 °C (marked with arrow 2). An endothermic peak with an onset temperature of 165 °C observed prior to milling had a similar onset temperature as the ITZ bulk material (165.6 °C). The onset of this peak was shifted from 165 °C to 152 °C during milling. The enthalpy of fusion decreased from 80.2 J/g to 60 J/g after 8 h of milling. Moreover, the enthalpy of the exothermic peak increased from 1 J/g after 5 h to 19.5 J/g after 8 h of milling.

**Figure 7:**
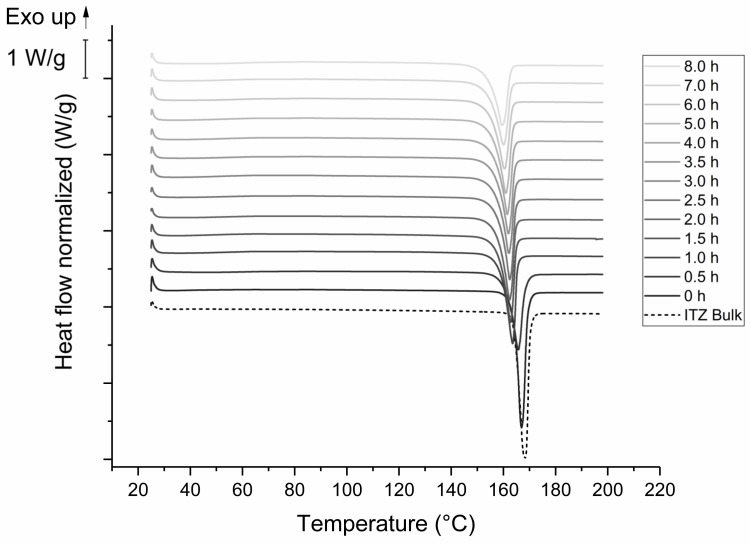
DSC-thermograms of F6 ITZ nanosuspension. Milling was carried out at 4 °C with 1500 rpm and milling beads sized 0.3–0.4 mm. Plots are shifted for a better illustration.

Figure 7 displays DSC-thermograms of formulation F6. The ITZ bulk material showed an endothermic peak with an onset temperature of 165.1 °C, which was also observable for the unprocessed ITZ suspension prior to milling. The onset is shifted to lower temperatures (e.g., to 154.8 °C after 8 h of milling), as already seen in the thermograms of formulation F2. The enthalpy decreased from 76 J/g to 61.2 J/g during the milling process.

#### 3.3.4. Impact of Concentration of Stabilizer on the Morphology

**Figure 8:**
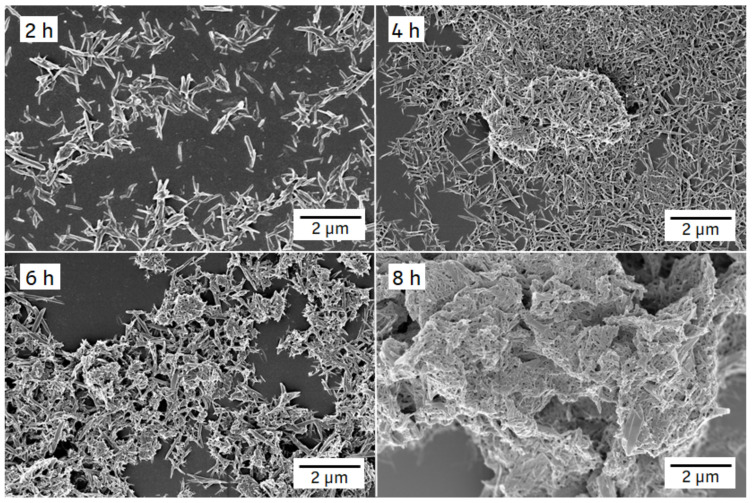
SEM images of F2 ITZ nanosuspension after different milling periods (indicated in the left upper corner of each image). Milling was carried out at 4 °C with 1500 rpm and milling beads sized 0.3–0.4 mm.

Figure 8 depicts SEM images of formulation F2. The ITZ crystals showed a conversion from the platelet-like shape of the jet-milled starting material (see Figure S8 in the Supplementary Materials) into a needle-like shape after 2 h of milling with almost no agglomerates. After 4 h, slight agglomeration occurred and further milling resulted in the formation of large agglomerates, as shown on the image after 8 h of milling.

**Figure 9:**
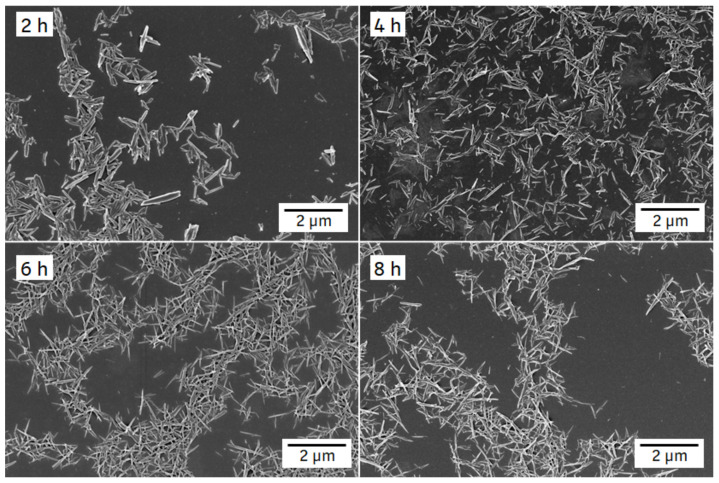
SEM images of F6 ITZ nanosuspension after different milling periods (indicated in the left upper corner of each image). Milling was carried out at 4 °C with 1500 rpm and milling beads sized 0.3–0.4 mm.

Figure 9 displays SEM images of formulation F6. Similar to formulation F2, the shape conversed from platelet to needle upon milling. However, unlike formulation F2, the particle size and shape of formulation F6 remained stable during the milling process and no agglomeration was observed up to 8 h.

#### 3.3.5. Impact of Concentration of Stabilizer on the Storage Stability

The particle size of both formulations, F2 and F6, were analyzed prior and after storage for 7 and 14 days at room temperature and under refrigerated conditions. Solid-state (XRPD/DSC) was investigated with samples milled for 2, 4, 6 and 8 h after storage at refrigerated conditions. The respective figures and further details can be found in the Supplementary Materials (Figures S2–S7).

Formulation F2 showed no significant change in particle size after 7 and 14 days of storage, regardless of the storage conditions and the duration of the milling process (0.07 ≤ *p* ≤ 0.98). Furthermore, an investigation of the behavior of the sample milled for 3 h after long-term (70 weeks) storage showed no significant particle size increase when stored at refrigerated conditions (*p* = 0.7). However, formulation F6 exhibited a statistically significant but very small increase in particle size after 7 days of storage (*p* < 0.001), regardless of the storage conditions and milling time applied, but no further significant change occurred after another 7 days of storage (*p* = 0.71 for refrigerated conditions; *p* = 0.29 for room temperature). Long-term storage investigation over 6 and 70 weeks at refrigerated conditions showed another slight increase between 2 and 6 weeks of storage (*p* = 0.05, rounded), but no further significant change occurred afterwards (*p* = 0.08).

The thermal behavior of formulation F2 showed differences in the thermogram after 7 days of storage compared to the initial thermogram directly after milling (0 d). The exothermic event at 90 °C that had occurred after milling for at least 6 h, as well as the endothermic event at about 60 °C after 8 h of milling, disappeared after storing the formulation. In contrast, the thermal behavior of formulation F6 remained unchanged after storage.

The XRPD solid-state properties of formulation F2 did not change during storage for the samples milled for 2 h, 4 h and 6 h, whereas an increase in peak intensity was observed after 7 days of storage of the samples milled for 8 h. A general reduction in peak height during prolonged milling could be seen for F2, as well as for F6. No qualitative change in solid-state was observed throughout storage for formulation F6.

## 4. Discussion

Our study revealed an increase in particle size for formulation F2 throughout 8 h of milling, which is accompanied by an increase in PDI. The zeta potential value decreased and implies an only moderately to insufficiently stabilized nanosuspension [24]. The solid–liquid interfacial area increases with increased milling time, resulting in increased Gibbs free energy and a thermodynamically unstable suspension [25]. If the stabilizer concentration is insufficient, van der Waals attraction may overcome repulsive forces of stabilizers and lead to agglomeration. Crystal growth has also been reported as reason for a particle size increase during or immediately after milling [26]; crystal growth can be excluded in our case as we could show progressive agglomeration over time. The time point of the change from a stable, crystalline nanoparticulate system to an instable, agglomerated system could be identified by DLS and zeta potential measurement. This observation was supported by SEM images and by the fact that a much higher SDS and PS80 concentration was needed to prevent agglomeration for the smallest ITZ particles exhibiting the highest surface area. A minimal concentration of 0.9% (*w*/*w*) HPC-SL, 0.14% (*w*/*w*) SDS and 0.07% (*w*/*w*) PS80 was necessary for a sufficient nanoparticle stabilization. Furthermore, the SDS concentration had a higher impact on the ITZ particle size than PS. Although PS80, as a non-ionic stabilizer (lower HLB value [27]), was not as potent as the ionic stabilizer SDS, it nevertheless was still necessary, especially for higher ITZ concentrations. The stabilizer concentration highly affected storage stability. The particle size significantly increased after 7 days of storage for formulation F6 (0.14% (*w*/*w*) SDS, 0.14% (*w*/*w*) PS80). A further particle growth was observed after 2 and 6 weeks, but then stopped. The use of high stabilizer concentrations obviously promoted Ostwald ripening during storage, as also already published by Verma et al. [8]. This group reported a significant increase in particle size upon 0 and 3 days of storage with high concentrations of stabilizer. On the contrary, the stabilizer concentration of formulation F2 was insufficient to prevent agglomeration during the milling process, but did not result in particle growth during storage.

Furthermore, it is well known that the milling process may impact the solid-state characteristics of an API [28]. Particles are exposed to a very high energy during milling, which can lead to plastic deformation, defects in the crystal structure, or on the surface, and the creation of (amorphous) areas of high-energy on a crystal, also known as mechanochemical activation [29,30]. The effect of the stabilizer concentration on the solid-state characteristics have not yet been investigated. Furthermore, nearly complete amorphization by media milling has not been reported yet. We could detect that the XRPD patterns of both formulations show an intensity reduction, as well as peak broadening, which is assumed to be caused by the smaller particle size or by crystal defects [22]. In general, reducing the particle size can result in XRPD peak broadening and halo formation, due to the loss of long-range crystalline order, but without complete transition to an amorphous form [31]. The reduced peak intensity in the case of formulation F2 indicated amorphization in addition. The XRPD pattern of the 8 h milled sample showed no complete amorphization as there are still crystal peaks evident; however, the content of amorphous ITZ seemed to be quite high. Complete amorphization might have occurred during further milling. The XRPD findings are in agreement with DSC studies of formulation F2. The endothermic event at around 62 °C is described in the literature as the glass transition temperature of ITZ [32]. Here, the glass transition was probably followed by relaxation due to mechanical stress of the sample. The following exothermic event at 87–90 °C could be attributed to some recrystallization of amorphous ITZ, as described in [33]. The crystalline ITZ melted at 165 °C, indicated by the endothermic event for the ITZ bulk material, which is also well reported in the literature [33]. The enthalpy of recrystallization increased over time and reached a maximum after 8 h of milling, indicated by the decreasing melting enthalpy. During the milling process, the onset temperatures of the melting temperature were shifted to lower temperatures, which might be related to crystal lattice defects [29], interaction with the polymer [34], or due to the small particle size of the nanoparticles [22]. In contrast to the aforementioned observations, no amorphous content was detected during milling of formulation F6. We conclude that the stabilization of the particle surface via stabilizers is stronger than the introduced mechanical conversion energy of crystalline into amorphous material by media milling. For F6, the XRPD pattern of the unprocessed ITZ suspension is similar to the ITZ bulk material and to already published reference samples [35]. Reduction of melting enthalpy and a shift of the onset temperature was also observed for the sufficient stabilized suspension F6, and is therefore related to the milling process. Furthermore, the changes in XRPD patterns upon the milling process are probably caused by the reduced particle size or crystal lattice defects, as described above.

During the process evaluation, a significant impact of the milling intensity on the particle size and the grinding limit could be shown: the higher the milling intensity, the earlier the grinding limit was reached.

Not only milling intensity, but also the size of the milling beads heavily affected the specific energy input, which determined the degree of comminution (i.e., particle size). The detailed kinetics of change in particle size in relation to the milling bead diameter has not yet been described to this extent for dual centrifugation. The smallest particle size could be obtained by using the smallest milling bead size. However, the smallest obtainable particle size could be only achieved by using a higher stabilizer concentration as smaller particles exhibit a larger specific surface area. The frequency of grinding contacts was highly increased for small milling beads due to the higher bead numbers per volume. This predominates the fact that the energy per contact is lower for smaller beads compared to larger beads [36]. The findings are in agreement with other studies investigating the effect of the milling bead size [29,37,38,39]. Remarkably, ITZ nanosuspensions with a relatively low particle size could be obtained even with the largest milling beads after 8 h of milling. This is attributed to the fact that the ZentriMix operates with a much higher specific energy compared to other bead mills, and the very long process duration. This assumption is supported by Hagedorn et al. [40], stating that the specific grinding energy of the ZentriMix is comparable or even higher to conventional agitator bead mills [40]. The effect of the temperature of the grinding chamber, respectively of the vial temperature, plays an important role during the formation of nanoparticles in terms of stability and particle growth, and has not been reported so far in the context of dual centrifugation [11,40]. Lower temperatures might be advantageous for very heat sensitive APIs, but could not be tested with the ZentriMix due to equipment inherent limitations. As also noted by Hagedorn et al. [11], the ZentriMix provides a material sparing approach for early-stage development with a high number (up to 40) of samples being processed simultaneously that offers benefits when compared to agitator bead mills. In addition, the option of using disposable vials is time saving, as tedious cleaning procedures as well as transferring the samples to a vessel for storage are inherently avoided. However, this process was found to require longer milling times when compared to experiments performed by Cerdeira et al. (2013), Li et al. (2018) or Bilgili et al. (2018), who reported a milling duration of about 60 min for ITZ [14,17,18], yielding similar particle size distributions. In addition, the batch size of the suspension usually is higher for wet stirred media milling, and 200–300 mL of sample can be processed with ITZ concentrations being comparable or even higher [14,16,17,18]. As only small amounts of materials are required (≤100 mg), we suggest performing early-stage experiments for development using the ZentriMix. As a next step, transferring the process to an agitator bead mill may be beneficial when larger batch sizes are required during later development stages. This approach has already been shown to be suitable for other APIs [40].

## 5. Conclusions

Media milling using the dual centrifugal principle of the ZentriMix proved to be a useful tool for small-scale formulation development of ITZ nanosuspensions, facilitated through the ability of running multiple samples within one process run, which is evaluated as being more efficient than reducing the duration of the individual processes. At a maximum speed of 1500 rpm, the grinding limit was primarily governed by the milling bead size, being 165–185 nm for bead sizes in between 0.2 and 1.00 mm at 3 h of milling, and 130 nm for bead sizes smaller than 0.2 mm already after 2 h of milling; in consequence, it is recommended as a process parameter to adjust the required particle size of the nanocrystals. Especially for insufficiently stabilized formulations, particle sizes of ITZ nanocrystals increased again after reaching the grinding limit upon further milling, accompanied with a similar trend for PDI and zeta potential. At insufficient protection of the nanocrystals surface, an increased mechanical energy intake resulted in an amorphization of the particles with pronounced agglomeration responsible for increasing PDI and particle size. Successful electrosteric stabilization, including long term stability, was found with the combination of HPC-SL (0.9% *w*/*w*), SDS (0.14% *w*/*w*) and PS80 (0.14% *w*/*w*) with SDS having the dominant impact on stabilization.

## Figures and Tables

**Table 1:** Overview of the parameters for the evaluation of the milling process. HPC-SL: hydroxypropyl cellulose, SDS: sodium dodecyl sulfate, PS80: polysorbate 80.

Purpose	Bead Size (mm)	Stabilizer Concentration (*w*/*w*)	Speed (rpm)	Temperature Inside the Milling Chamber (°C)
Speed evaluation	0.3–0.4	0.9% HPC-SL, 0.14% SDS, 0.14% PS80	500	4
			1500	
Temperature evaluation	0.3–0.4	0.9% HPC-SL, 0.14% SDS, 0.14% PS80	1500	4
				−10
Bead size evaluation	0.1–0.2	0.9% HPC-SL, 0.28% SDS, 0.28% PS80	1500	4
	0.3–0.4	0.9% HPC-SL, 0.14% SDS, 0.14% PS80		
	0.4–0.6			
	0.6–0.8			
	0.8–1.0			
	1.2–1.4			
	1.6–1.8			
	2.0–2.5			
	2.6–3.3			
Stabilizer impact on CMA	0.3–0.4	0.9% HPC-SL, 0.14% SDS, 0.14% PS80	1500	4
		0.9% HPC-SL, 0.07% SDS, 0.07% PS80		

**Table 2:** Input variables and response parameters of the statistical evaluation of the stabilizer concentrations after 8 h of milling. HPC-SL concentration was kept constant in all experiments (0.9% (*w*/*w*)). PDI: Polydispersity index.

Formulation	Input		Response	
	SDS Concentration (% *w*/*w*)	PS80 Concentration (% *w*/*w*)	Z-Average (nm)	PDI
F1	0	0.14	1443	0.19
			1465	0.30
			1378	0.28
F2	0.07	0.07	4935	0.73
			5447	0.79
			5609	1.00
F3	0.07	0.14	2254	0.29
			2201	0.58
			2464	0.24
F4	0.14	0	221	0.24
			232	0.26
			226	0.31
F5	0.14	0.07	162	0.22
			168	0.22
			167	0.22
F6	0.14	0.14	165	0.23
			165	0.22
			166	0.22

**Table 3:** Summary of fit for the applied model. R^2^: measure of fit, Q^2^: estimation of precision for the prediction, N: number of trials.

R^2^	R^2^ Adj.	Q^2^	N	Model Validity	Reproducibility
0.9953	0.9938	0.99	18	0.94	0.99

**Table 4:** Overview of the bead diameters, density, bead volume and the number of beads used per run.

Bead Diameter (mm)	Bead Density (g/cm^3^)	Bead Volume of 5 g Beads (cm^3^)	Theoretical Number of Beads in 5 g
0.1–0.2	6	0.83	471,570
0.3–0.4			37,121
0.4–0.6			12,732
0.6–0.8			4640
0.8–1.0			1592
1.2–1.4			724
1.6–1.8			324
2.0–2.5			131
2.6–3.3			59

## Data Availability

Not applicable.

## Supplementary Materials

The following supporting information can be downloaded at: https://www.mdpi.com/article/10.3390/pharmaceutics14081528/s1, Figure S1: Effect of milling bead size on the polydispersity index (PDI) of ITZ particles (relating to F6); Figure S2: Particle sizes of formulation F2 stored at refrigerated conditions (A) and room temperature (B) for different storage periods; Figure S3: Particle sizes of formulation F6 stored at refrigerated conditions (A) and room temperature (B) after different storage periods; Figure S4: DSC thermograms of F2 ITZ nanosuspension after storage at refrigerated conditions for storage periods of 0, 7 and 14 days; Figure S5: DSC thermograms of F6 ITZ nanosuspension after storage at refrigerated conditions for different storage periods of 0, 7 and 14 days; Figure S6: XRPD patterns of F2 ITZ nanosuspension after storage for different storage periods of 0, 7 and 14 days at refrigerated conditions; Figure S7: XRPD patterns of the F6 ITZ nanosuspension after storage for different storage periods of 0, 7 and 14 days at refrigerated conditions. Milling was carried out at 4 °C with 1500 rpm and milling beads sized 0.3–0.4 mm; Figure S8: SEM image of jet-milled (JM) ITZ particles used as starting material for nanomilling.

## References

[1] Williams H.D., Trevaskis N.L., Charman S.A., Shanker R.M., Charman W.N., Pouton C.W., Porter C.J.H. (2013). Strategies to Address Low Drug Solubility in Discovery and Development. Pharmacol. Rev..

[2] Noyes A., Whitney W.R. (1897). The Rate of Solution of Solid Substances in Their Own Solutions. J. Am. Chem. Soc..

[3] Bobo D., Robinson K.J., Islam J., Thurecht K.J., Corrie S.R. (2016). Nanoparticle-Based Medicines: A Review of FDA-Approved Materials and Clinical Trials to Date. Pharmaceut. Res..

[4] Li M., Azad M., Davé R., Bilgili E. (2016). Nanomilling of Drugs for Bioavailability Enhancement: A Holistic Formulation-Process Perspective. Pharmaceutics.

[5] Rabinow B.E. (2004). Nanosuspensions in Drug Delivery. Nat. Rev. Drug Discov..

[6] Ostwald W. (1897). Studien Über Die Bildung Und Umwandlung Fester Körper. Z. Phys. Chem..

[7] Li J., Wang Z., Zhang H., Gao J., Zheng A. (2020). Progress in the Development of Stabilization Strategies for Nanocrystal Preparations. Drug Deliv..

[8] Verma S., Kumar S., Gokhale R., Burgess D.J. (2011). Physical Stability of Nanosuspensions: Investigation of the Role of Stabilizers on Ostwald Ripening. Int. J. Pharm..

[9] Derjaguin B.V., Landau L. (1941). Theory of the Stability of Strongly Charged Lyophobic Sols and of the Adhesion of Strongly Charged Particles in Solution of Electrolytes. Acta Physicochim. USSR.

[10] Verwey E.J.W. (1947). Theory of the Stability of Lyophobic Colloids. J. Phys. Colloid Chem..

[11] Hagedorn M., Bögershausen A., Rischer M., Schubert R., Massing U. (2017). Dual Centrifugation—A New Technique for Nanomilling of Poorly Soluble Drugs and Formulation Screening by an DoE-Approach. Int. J. Pharm..

[12] Six K., Daems T., de Hoon J., Hecken A.V., Depre M., Bouche M.-P., Prinsen P., Verreck G., Peeters J., Brewster M.E. (2005). Clinical Study of Solid Dispersions of Itraconazole Prepared by Hot-Stage Extrusion. Eur. J. Pharm. Sci..

[13] Bhakay A., Merwade M., Bilgili E., Dave R.N. (2011). Novel Aspects of Wet Milling for the Production of Microsuspensions and Nanosuspensions of Poorly Water-Soluble Drugs. Drug Dev. Ind. Pharm..

[14] Cerdeira A.M., Mazzotti M., Gander B. (2013). Formulation and Drying of Miconazole and Itraconazole Nanosuspensions. Int. J. Pharm..

[15] Kumar S., Jog R., Shen J., Zolnik B., Sadrieh N., Burgess D.J. (2015). In Vitro and In Vivo Performance of Different Sized Spray-Dried Crystalline Itraconazole. J. Pharm. Sci..

[16] Azad M., Moreno J., Bilgili E., Davé R. (2016). Fast Dissolution of Poorly Water Soluble Drugs from Fluidized Bed Coated Nanocomposites: Impact of Carrier Size. Int. J. Pharmaceut..

[17] Li M., Suriel I., Vekaria J., Proske J., Orbe P., Armani M., Dave R.N., Bilgili E. (2018). Impact of Dispersants on Dissolution of Itraconazole from Drug-Loaded, Surfactant-Free, Spray-Dried Nanocomposites. Powder Technol..

[18] Bilgili E., Rahman M., Palacios D., Arevalo F. (2018). Impact of Polymers on the Aggregation of Wet-Milled Itraconazole Particles and Their Dissolution from Spray-Dried Nanocomposites. Adv. Powder Technol..

[19] Knieke C., Sommer M., Peukert W. (2009). Identifying the Apparent and True Grinding Limit. Powder Technol..

[20] Howe H.W., Warriner J.J., Cook A.M., Coguill S.L., Farrar L.C. (2011). Method for Resonant-Vibratory Mixing 2011. U.S. Patent.

[21] Tanaka R., Takahashi N., Nakamura Y., Hattori Y., Ashizawa K., Otsuka M. (2016). Verification of the Mixing Processes of the Active Pharmaceutical Ingredient, Excipient and Lubricant in a Pharmaceutical Formulation Using a Resonant Acoustic Mixing Technology. RSC Adv..

[22] Peltonen L. (2018). Practical Guidelines for the Characterization and Quality Control of Pure Drug Nanoparticles and Nano-Cocrystals in the Pharmaceutical Industry. Adv. Drug Deliv. Rev..

[23] Keck C.M. (2006). Cyclosporine Nanosuspensions: Optimised Size Characterisation & Oral Formulations. Ph.D. Thesis.

[24] Kumar A., Dixit C.K. (2017). Advances in Nanomedicine for the Delivery of Therapeutic Nucleic Acids.

[25] Gibbs J.W. (1873). A Method of Geometrical Representation of the Thermodynamic Properties by Means of Surfaces. Trans. Conn. Acad. Arts Sci..

[26] Bitterlich A., Laabs C., Krautstrunk I., Dengler M., Juhnke M., Grandeury A., Bunjes H., Kwade A. (2015). Process Parameter Dependent Growth Phenomena of Naproxen Nanosuspension Manufactured by Wet Media Milling. Eur. J. Pharm. Biopharm..

[27] Rowe R.C., Sheskey P.J., Weller P.J., Rowe R.C. (2003). Handbook of Pharmaceutical Excipients.

[28] Kumar S., Burgess D.J. (2014). Wet Milling Induced Physical and Chemical Instabilities of Naproxen Nano-Crystalline Suspensions. Int. J. Pharm..

[29] Peltonen L., Hirvonen J. (2010). Pharmaceutical Nanocrystals by Nanomilling: Critical Process Parameters, Particle Fracturing and Stabilization Methods. J. Pharm. Pharmacol..

[30] Colombo I., Grassi G., Grassi M. (2009). Drug Mechanochemical Activation. J. Pharm. Sci..

[31] Deng Z., Xu S., Li S. (2008). Understanding a Relaxation Behavior in a Nanoparticle Suspension for Drug Delivery Applications. Int. J. Pharm..

[32] Six K., Verreck G., Peeters J., Binnemans K., Berghmans H., Augustijns P., Kinget R., Mooter G.V. (2001). den Investigation of Thermal Properties of Glassy Itraconazole: Identification of a Monotropic Mesophase. Thermochim. Acta.

[33] Yang W., Johnston K.P., Williams R.O. (2010). Comparison of Bioavailability of Amorphous versus Crystalline Itraconazole Nanoparticles via Pulmonary Administration in Rats. Eur. J. Pharm. Biopharm..

[34] Sharma P., Denny W.A., Garg S. (2009). Effect of Wet Milling Process on the Solid State of Indomethacin and Simvastatin. Int. J. Pharm..

[35] Verreck G., Six K., den Mooter G.V., Baert L., Peeters J., Brewster M.E. (2003). Characterization of Solid Dispersions of Itraconazole and Hydroxypropylmethylcellulose Prepared by Melt Extrusion—Part I. Int. J. Pharm..

[36] Kwade A. (1999). Determination of the Most Important Grinding Mechanism in Stirred Media Mills by Calculating Stress Intensity and Stress Number. Powder Technol..

[37] Gao M., Forssberg E. (1995). Prediction of Product Size Distributions for a Stirred Ball Mill. Powder Technol..

[38] Kwade A., Schwedes J. (1997). Wet Comminution in Stirred Media Mills. Kona Powder Part. J..

[39] Li M., Alvarez P., Bilgili E. (2017). A Microhydrodynamic Rationale for Selection of Bead Size in Preparation of Drug Nanosuspensions via Wet Stirred Media Milling. Int. J. Pharm..

[40] Hagedorn M., Liebich L., Bögershausen A., Massing U., Hoffmann S., Mende S., Rischer M. (2019). Rapid Development of API Nano-Formulations from Screening to Production Combining Dual Centrifugation and Wet Agitator Bead Milling. Int. J. Pharm..

