# Supplementary material for: Itraconazole Nanosuspensions via Dual Centrifugation Media Milling: Impact of Formulation and Process Parameters on Particle Size and Solid-State Conversion as Well as Storage Stability
Source: Pharmaceutics. 2022 Jul 22;14(8):1528. doi: 10.3390/pharmaceutics14081528 (PMC9332252; doi:10.3390/pharmaceutics14081528)
Supplement: Supplementary file 1 [file pharmaceutics-14-01528-s001.zip › pharmaceutics-1789617-supplementary.pdf]

Supplementary material to:

Itraconazole nanosuspensions via dual centrifugation media milling: impact of formulation and process parameters on particle size and solid-state conversion as well as storage stability

Ann-Cathrin Willmann[1,2], Kai Berkenfeld[2], Thilo Faber[2], Herbert Wachtel[3], Georg Boeck[4], Karl G. Wagner[2]*

[1] Pharmaceutical Development, Boehringer Ingelheim Pharma GmbH & Co. KG, Birkendorfer Straße 65, 88397 Biberach, Germany

[2] Department of Pharmaceutical Technology, University of Bonn, Gerhard-Domagk-Straße 3, 53121 Bonn, Germany

[3] Device Development, Boehringer Ingelheim Pharma GmbH & Co. KG, Binger Straße 173, 55216 Ingelheim am Rhein

[4] Department Discovery Research, Boehringer Ingelheim RCV GmbH & Co KG, Dr.-Boehringer-Gasse 5-11, 1121 Vienna, Austria

* Prof. Dr. Karl Gerhard Wagner, Department of Pharmaceutical Technology, University of Bonn, Ger-hard-Domagk-Straße 3, 53121 Bonn, Germany, Phone: +49 228 73 5271, E-mail address: karl.wagner@uni-bonn.de

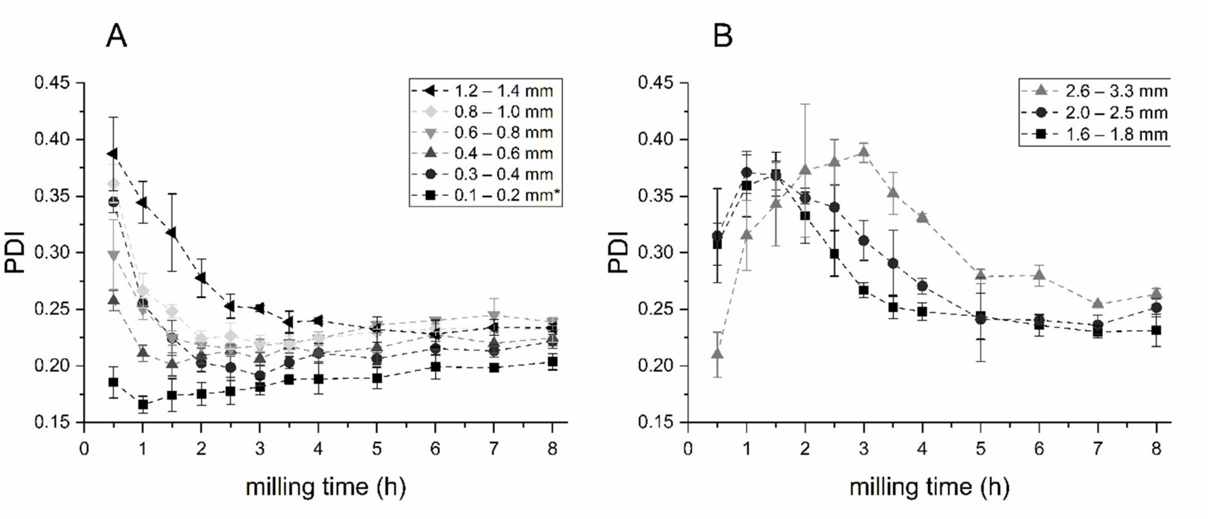

Figure S1. Effect of milling bead size on the polydispersity index (PDI) of ITZ particles (relating to F6). Values are expressed as mean ± SD, n = 3. Milling was carried out at 4°C with 1500 rpm. *Bead size 0.1 - 0.2 mm: stabilizer concentration had to be changed to 0.9% (w/w) HPC-SL, 0.28%(w/w) PS80 and 0.28% (w/w) SDS (see chapter 4). Data are shown in two figures (A & B) for better readability.

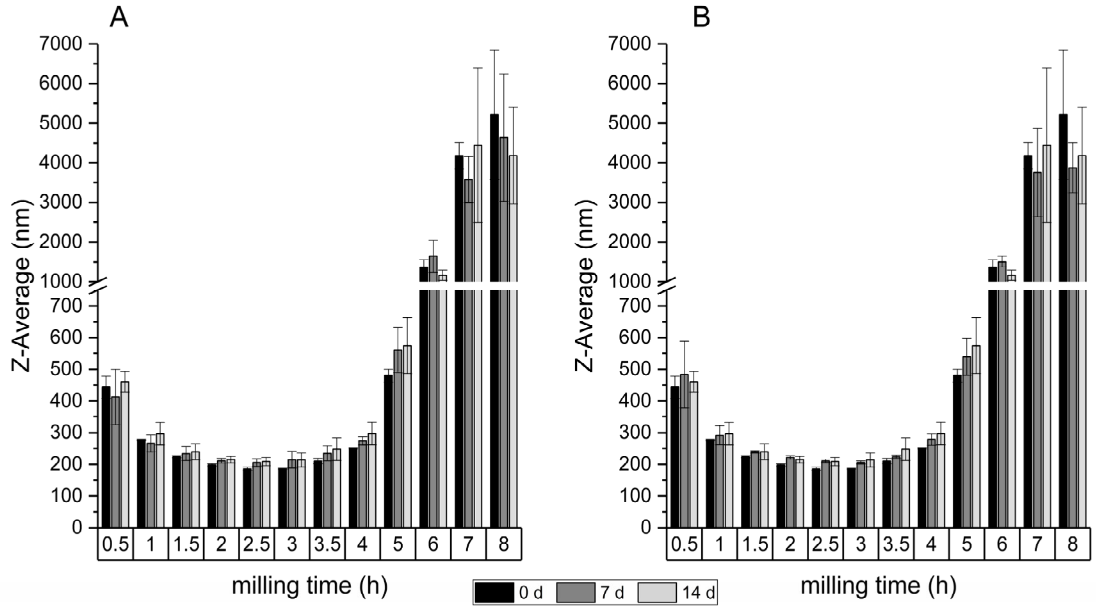

Figure S2. Particle sizes of formulation F2 stored at refrigerated conditions (A) and room temperature (B) for different storage periods. Values are expressed as mean ± SD, n = 3. Milling was carried out at 4°C with 1500 rpm and milling beads sized 0.3 – 0.4 mm.

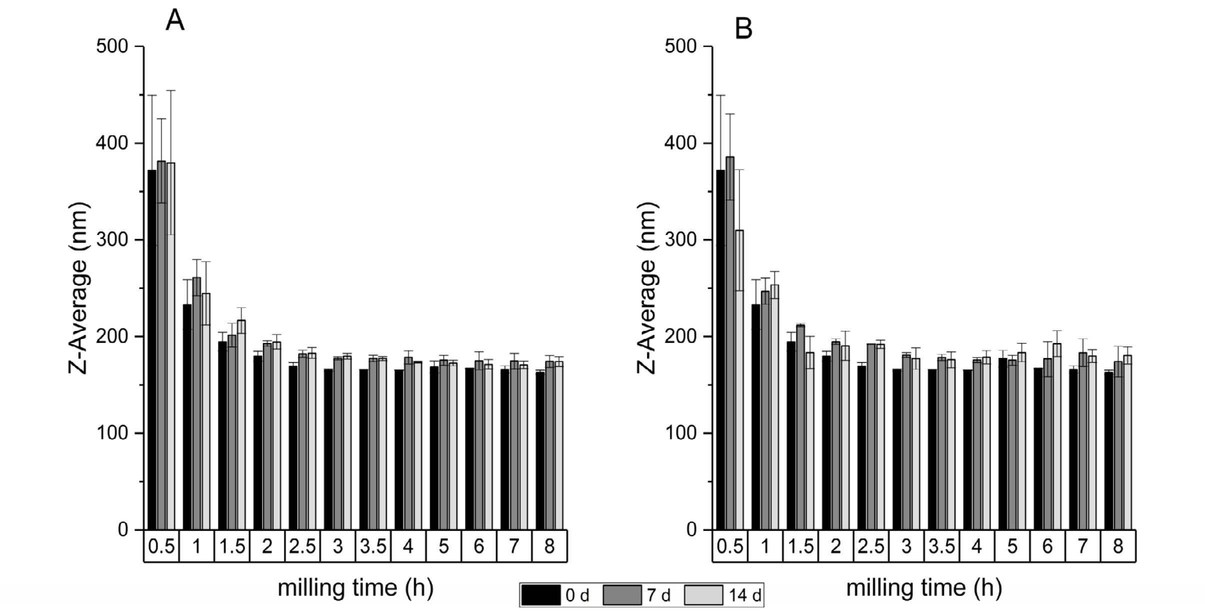

Figure S3. Particle sizes of formulation F6 stored at refrigerated conditions (A) and room temperature (B) after different storage periods. Values are expressed as mean ± SD, n = 3. Milling was carried out at 4°C with 1500 rpm and milling beads sized 0.3 – 0.4 mm.

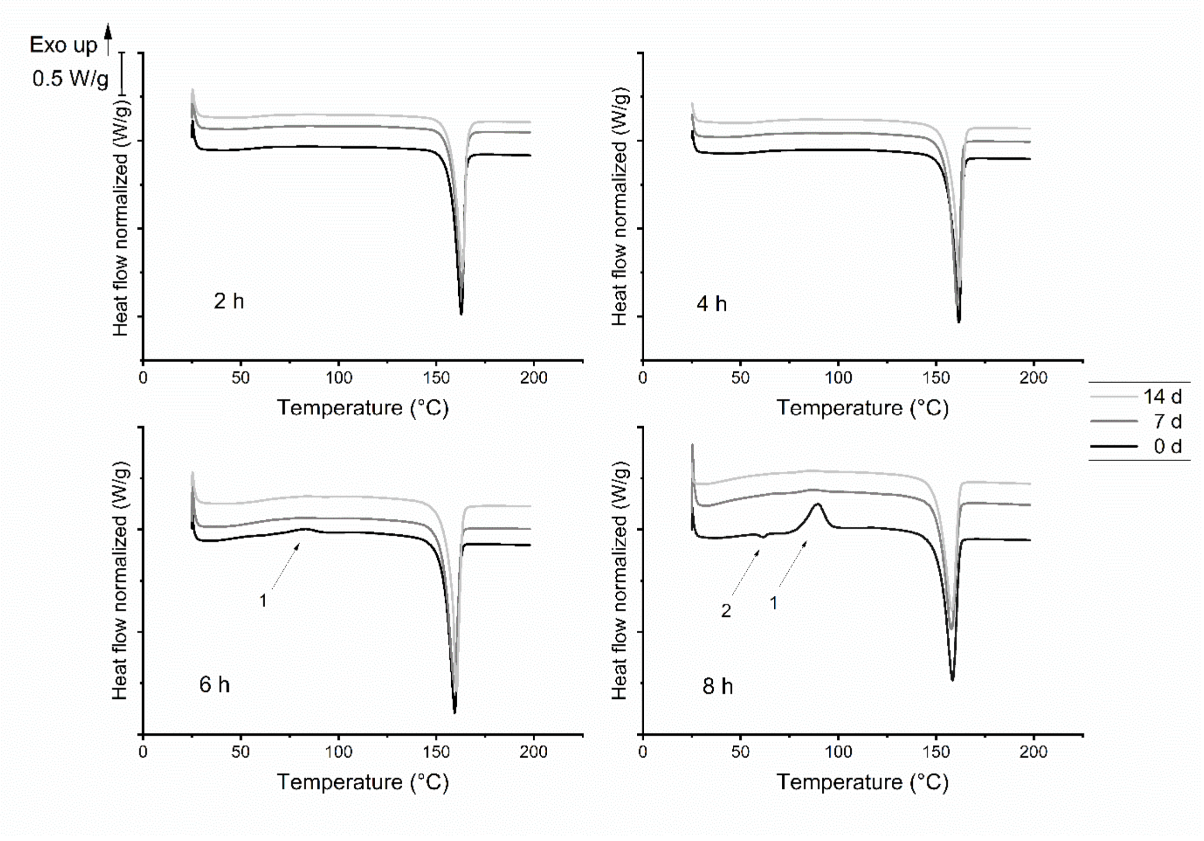

Figure S4. DSC thermograms of F2 ITZ nanosuspension after storage at refrigerated conditions for storage periods of 0, 7, and 14 days. Milling was carried out at 4°C with 1500 rpm and milling beads sized 0.3 – 0.4 mm. Milling time is indicated in the lower left corner of each figure. Plots are shifted for a better illustration. Arrow 1 marks an exothermic event, arrow 2 marks an endothermic event.

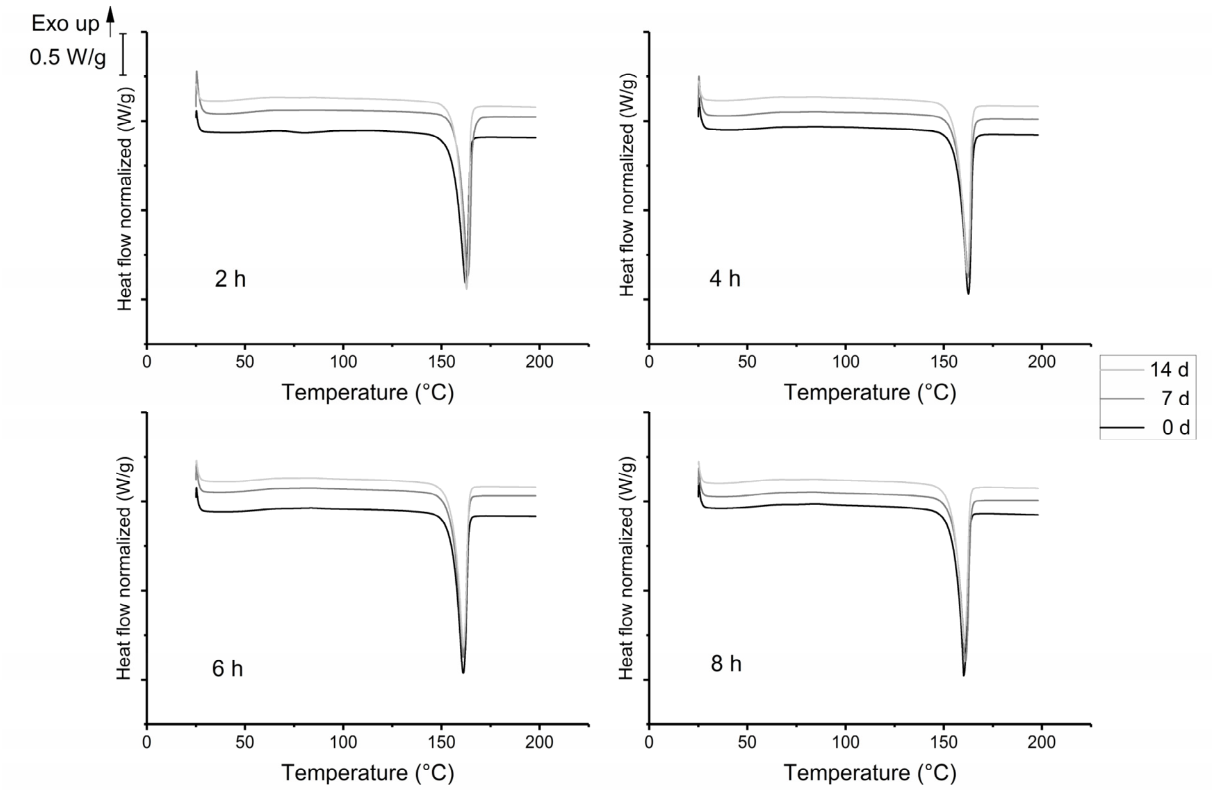

Figure S5. DSC thermograms of F6 ITZ nanosuspension after storage at refrigerated conditions for different storage periods of 0, 7, and 14 days. Milling was carried out at 4°C with 1500 rpm and milling beads sized 0.3 – 0.4 mm. Milling time is indicated in the lower left corner of each figure. Plots are shifted for a better illustration.

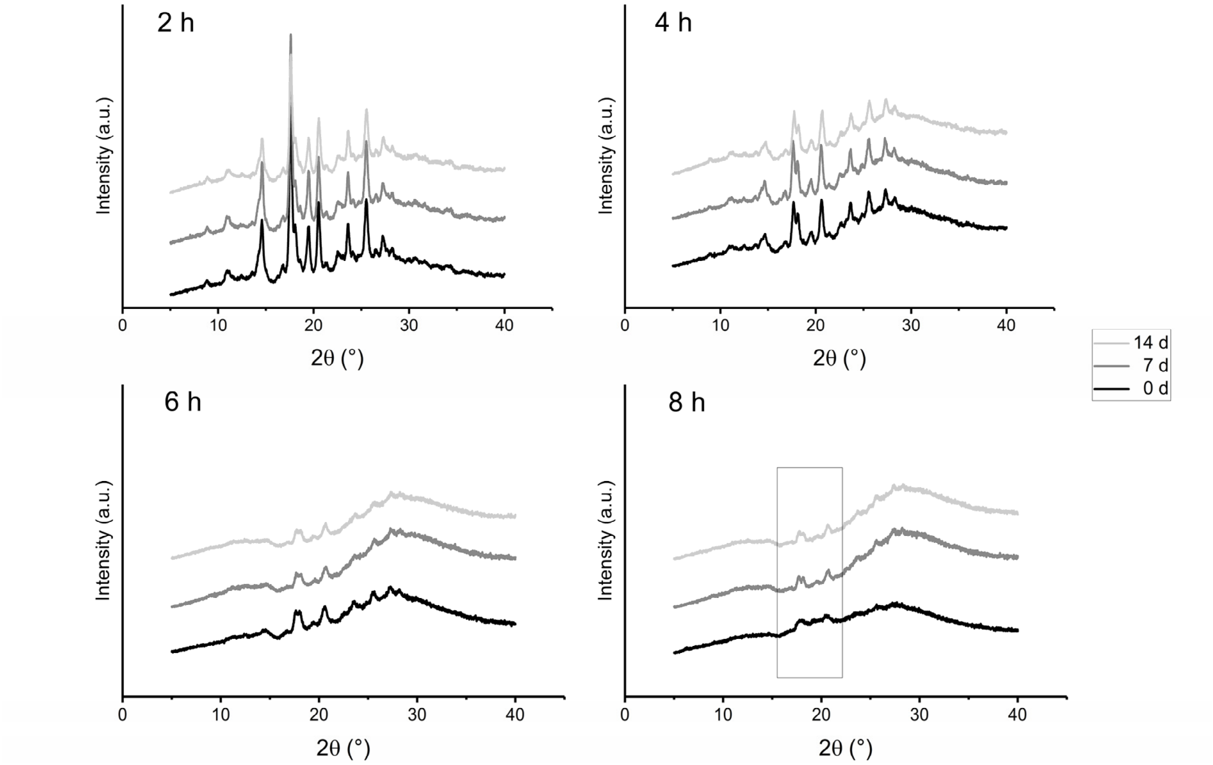

Figure S6. XRPD patterns of F2 ITZ nanosuspension after storage for different storage periods of 0, 7, and 14 days at refrigerated conditions. Milling was carried out at 4°C with 1500 rpm and milling beads sized 0.3 – 0.4 mm. Milling time is indicated in the upper left corner of each figure. Plots are shifted for better a illustration. Marked area: increase in peak intensity.

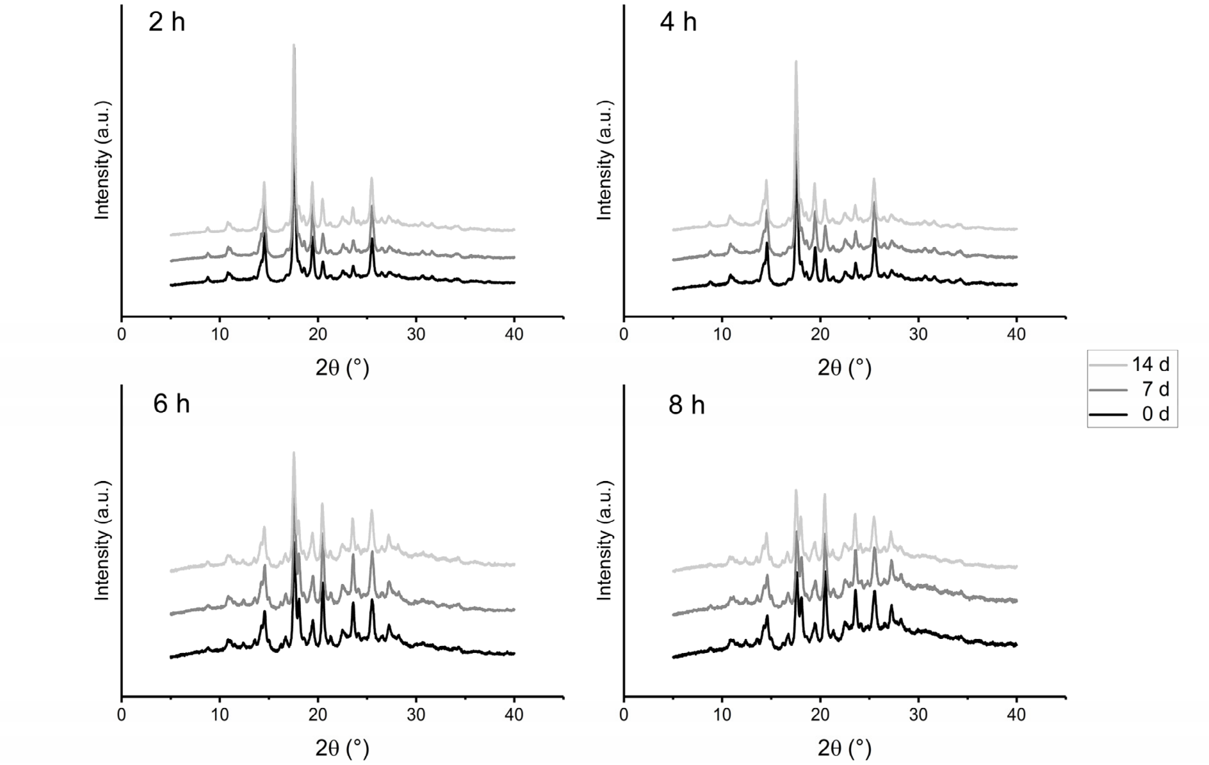

Figure S7. XRPD patterns of the F6 ITZ nanosuspension after storage for different storage periods of 0, 7, and 14 days at refrigerated conditions. Milling was carried out at 4°C with 1500 rpm and milling beads sized 0.3 – 0.4 mm. Milling time is indicated in the upper left corner of each figure. Plots are shifted for a better illustration.

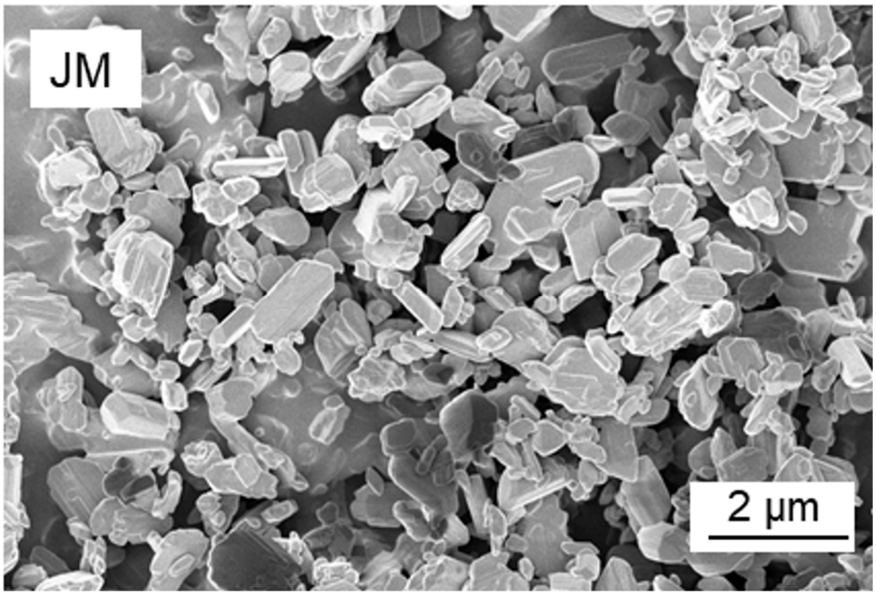

Figure S8. SEM image of jet-milled (JM) ITZ particles used as starting material for nanomilling.
